# Identification of long noncoding RNAs reveals the effects of dinotefuran on the brain in *Apis mellifera* (Hymenopptera: Apidae)

**DOI:** 10.1186/s12864-021-07811-y

**Published:** 2021-07-03

**Authors:** Minjie Huang, Jie Dong, Haikun Guo, Minghui Xiao, Deqian Wang

**Affiliations:** 1 Institute of Animal Husbandry and Veterinary Science, Zhejiang Academy of Agricultural Sciences, Hangzhou, China; 2 Institute of Quality and Standard for Agro-Products, Zhejiang Academy of Agricultural Sciences, Hangzhou, China; 3 State Key Laboratory of Subtropical Silviculture, Zhejiang A&F University, Hangzhou, China

**Keywords:** lncRNA, Dinotefuran, Brain, *Apis mellifera*, Neonicotinoid insecticide

## Abstract

**Background:**

Dinotefuran (CAS No. 165252–70-0), a neonicotinoid insecticide, has been used to protect various crops against invertebrate pests and has been associated with numerous negative sublethal effects on honey bees. Long noncoding RNAs (lncRNAs) play important roles in mediating various biological and pathological processes, involving transcriptional and gene regulation. The effects of dinotefuran on lncRNA expression and lncRNA function in the honey bee brain are still obscure.

**Results:**

Through RNA sequencing, a comprehensive analysis of lncRNAs and mRNAs was performed following exposure to 0.01 mg/L dinotefuran for 1, 5, and 10 d. In total, 312 lncRNAs and 1341 mRNAs, 347 lncRNAs and 1458 mRNAs, and 345 lncRNAs and 1155 mRNAs were found to be differentially expressed (DE) on days 1, 5 and 10, respectively. Gene set enrichment analysis (GSEA) indicated that the dinotefuran-treated group showed enrichment in carbohydrate and protein metabolism and immune-inflammatory responses such as glycine, serine and threonine metabolism, pentose and glucuronate interconversion, and Hippo and transforming growth factor-β (TGF-β) signaling pathways. Moreover, the DE lncRNA TCONS_00086519 was shown by fluorescence in situ hybridization (FISH) to be distributed mainly in the cytoplasm, suggesting that it may serve as a competing endogenous RNA and a regulatory factor in the immune response to dinotefuran.

**Conclusion:**

This study characterized the expression profile of lncRNAs upon exposure to neonicotinoid insecticides in young adult honey bees and provided a framework for further study of the role of lncRNAs in honey bee growth and the immune response.

**Supplementary Information:**

The online version contains supplementary material available at 10.1186/s12864-021-07811-y.

## Background

Neonicotinoid pesticides are the most widely used and effective insecticides against multiple herbivorous insects worldwide [1]. Neonicotinoid pesticides are systemic insecticides that act as a nicotinic acetylcholine receptor (nAChR) agonists, which make insects extremely prone to death, mainly by binding to nAChRs [2]. However, neonicotinoid insecticides can have negative effects on the survival and health of nontarget beneficial insects, such as honey bees, which are important pollinators. Forager honey bees are exposed to neonicotinoid pesticides by collecting contaminated pollen and nectar, which are then stored in beehives [3]. Therefore, nurses, young in-hive honey bees and larvae are also exposed to neonicotinoid pesticides through the acquisition of nectar, pollen and bee bread. Sublethal doses of neonicotinoid pesticides have been shown to impair the development of the brain and mushroom bodies [4], affect olfactory learning and memory abilities [5, 6], and disrupt navigation by forager honey bees [7]. Neonicotinoid accumulation in the bee brain disrupts circadian rhythmicity and impairs sleep in many bees [8]. Dinotefuran belongs to the third -generation of neonicotinoid insecticides and shows broad-spectrum, systemic insecticidal activity [9], which is highly toxic to *A. mellifera* [10]. Dinotefuran residues have been detected in nectar, pollen and bee bread, with measured residues in pollen of approximately 147 ng/g [11]. Studies have shown that treatment with a sublethal dose of dinotefuran affects olfaction, octopamine concentrations, learning and homing ability in honey bees [12, 13].

Long noncoding RNAs (lncRNAs) are a class of nonprotein-coding RNAs of more than 200 nucleotides in length that have been found in numerous species [14]. It is increasingly clear that lncRNAs act in association with other molecules as regulators in several different physiological processes, immune responses, the pathogenesis of various human diseases and other biological processes [15–17]. A series of studies have shown that lncRNAs play important roles in growth, development, caste differentiation, and innate immunity in honey bees. The noncoding RNA *Nb-1* in the worker brain is involved in the synthesis and secretion of juvenile hormone, indicating a potential role in social behaviors [18]. Fernanda et al. first observed that lncov1 was overexpressed in worker ovaries and demonstrated an expression peak coinciding with the onset of autophagic cell death [19]. LncRNAs in honey bees show tissue-specific expression and are preferentially expressed in ovarian and fat body tissues, suggesting that they are associated with biological and hormone signaling pathways and various diseases of honey bees [20]. Previous studies have shown the involvement of lncRNAs in the regulation of host-pathogen interactions, such as the responses to *Nosema ceranae* infection [21], or *Ascospheara apis* infection [22] and virus infection in honey bees [23]. However, the potential role of lncRNAs in the response of *A. mellifera* to dinotefuran has not been reported.

In the present study, we performed RNA-seq to detect the profile of lncRNAs in the brains of young adult bees exposed to sublethal doses of dinotefuran and identified differentially expressed (DE) transcripts. To further investigate the function of DE lncRNAs in the honey bee brain, the distribution of the highly expressed and DE lncRNA TCONS_00086519 was determined through subcellular localization. The results not only lay a foundation for elucidating the molecular mechanisms underlying the effects of exposure to neonicotinoid insecticides, but also offer a beneficial resource for the functional study of key dinotefuran-responsive lncRNAs in further studies.

## Results

### Characterization of the brain tissue transcriptome

Approximately 100 million raw reads per sample were obtained from the 18 cDNA libraries (Additional file 1). After removing adaptor sequences and low-quality reads, more than 82.35% of clean reads were mapped to the *A. mellifera* reference genome using Hisat2 (Table S1), and approximately 74% of clean reads were aligned with unique loci. Most reads were aligned to exon regions while nearly 2.37–6.54% and 12.52–23.73% originated from introns and intergenic regions, respectively (Additional file 1). However, approximately 10% of the clean reads were mapped outside of annotated regions.

### Known mRNA profiling in honey bee brains

**Fig. 1:**
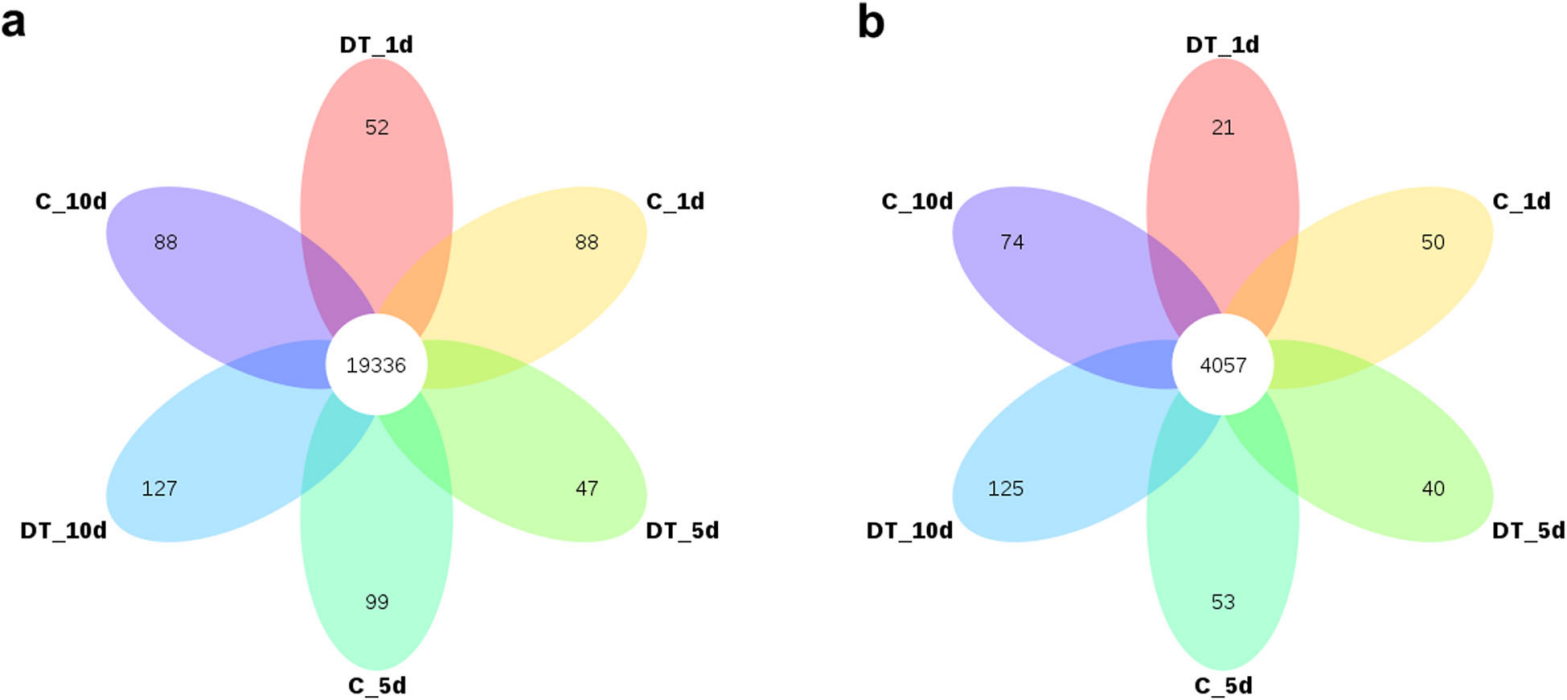
Identification of coding transcripts (mRNAs) and lncRNAs. (**a**) Venn diagram of mRNAs identified between dinotefuran-treated bees and control bees at three experimental time points; (**b**) Venn diagram of lncRNAs identified between dinotefuran-treated bees and control bees at three experimental time points

In total, 19,837 known mRNAs were identified according to the reference honey bee genome. Most of these mRNAs (19,336) were expressed in both the dinotefuran-treated and untreated control groups, while 275 were specifically expressed in the dinotefuran-treated group, and 226 were specifically expressed in the control group (Fig. 1a). However, most of the treatment specific mRNAs exhibited a low expression level. Transcripts from the major royal jelly protein family (Mrjp1, Mrjp2, Mrjp3), chemosensory proteins (CSP1, CSP3), apidermin (APD-2, APD-3) and NADH dehydrogenase subunit 1(ND1) were the most abundant at the three examined time points (Additional file 2). Among the identified transcripts, immune-related transcripts such as defensin 1 (Def1), cyclooxygenase 1 (COX1), abaecin (LOC406144) and hymenoptaecin (LOC406142) were highly expressed in bee brains. Gene ontology (GO) analysis showed that the highly expressed transcripts (FPKM > 1000) were annotated with cellular component-related terms such as extracellular region and ribosome, biological process-related terms such as defense response to bacterium and innate immune response, and molecular function-related terms such as structural constituent of ribosome, suggesting that dinotefuran treatment may cause high energy consumption and an immune response.

### Identification of lncRNA

After transcript assembly, lncRNAs were defined according to a series of filtering criteria (Additional file 3) using Cuffmerge and Cuffcompare software. Through coding potential analysis with Coding Potential Calculator 2 (CPC2), PfamScan and Coding-Non-Coding Index (CNCI), 6824 lncRNA transcripts were identified in the honey bee brain transcriptome. Among these transcripts, 186 were specifically expressed in the dinotefuran-treated group, and 177 were specifically expressed in the control group (Fig. 1b). Although approximately 90% of lncRNA transcripts exhibited a low expression level (PFKM < 1), transcripts such as TCONS_00024915 and TCONS_00086477 were highly expressed in the honey bee brain (Additional file 4), suggesting that these lncRNAs may play a specific role in in the effects of neonicotinoid insecticides on honey bees.

### Comparative features of mRNAs and lncRNAs

**Fig. 2:**
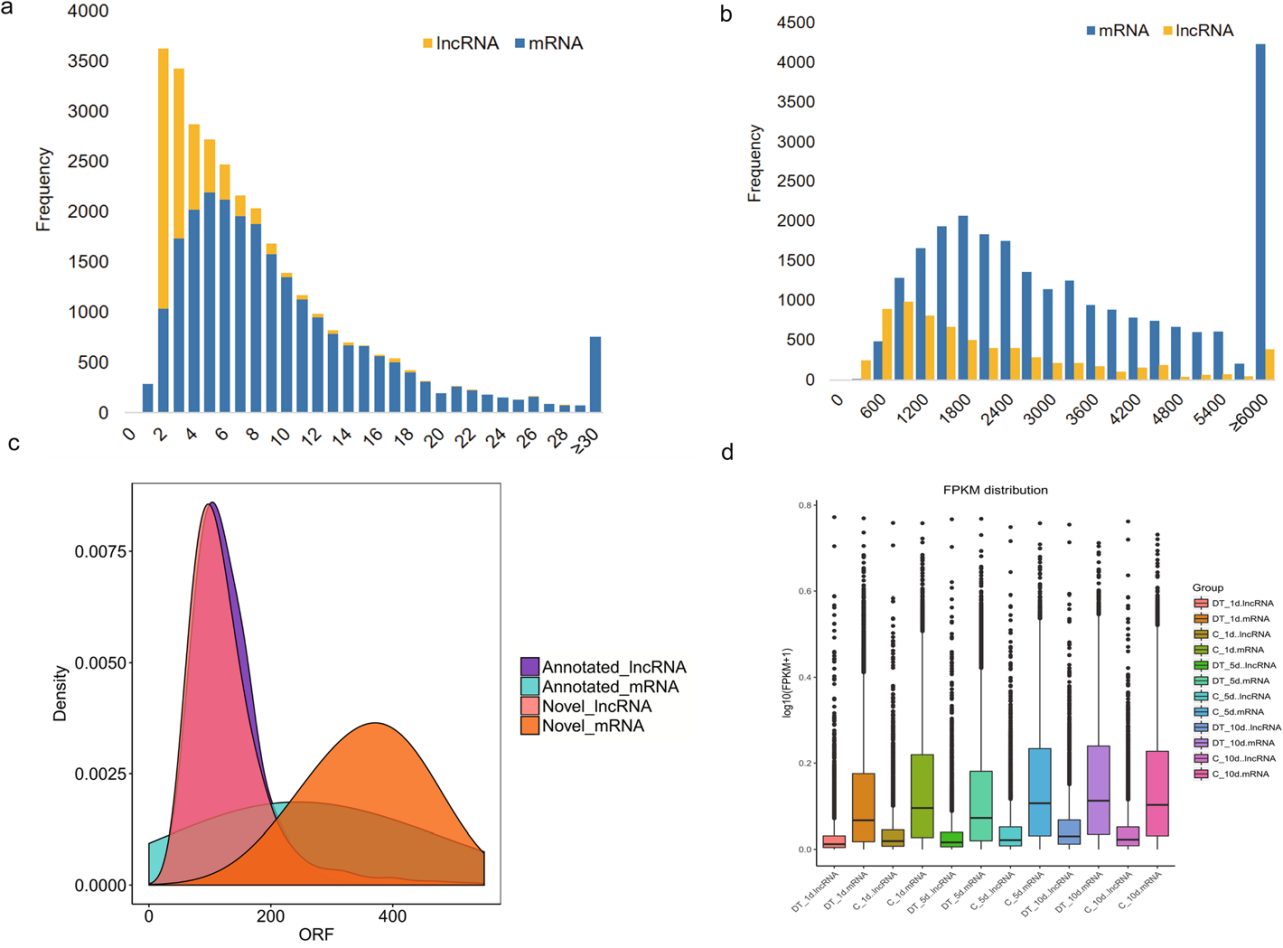
Comparative analysis of mRNA and lncRNA characteristics. (**a**) Exon number distribution in mRNAs and lncRNAs; (**b**) length distribution of the mRNAs and lncRNAs; (**c**) ORF length density distribution in mRNAs and lncRNAs; (**d**) expression level distribution among mRNAs and lncRNAs identified between dinotefuran-treated bees and control bees

Most lncRNAs identified in this study contained fewer exons (Fig. 2a) and were shorter in length than mRNAs (Fig. 2b), which is consistent with the results of previous studies [24, 25]. More than 63.34% of the lncRNAs contained two to three exons, while only 11.32% of the mRNAs contained two to three exons. Approximately 40% of the lncRNAs ranged from 300 to 1200 bp in length, which was much higher than the percentage of mRNAs (14%) identified in this size range. Based on reference gene structures and predictions from EMBOSS explorer (http://emboss.bioinformatics.nl/), we found that the open reading frames (ORFs) of the mRNAs were much longer than those of the lncRNAs in both annotated and novel transcripts (Fig. 2c). In addition, the expression levels of the lncRNAs were found to be much lower than those of the mRNAs in each library (Fig. 2d).

### Differential expression analysis

Differential expression analysis of the lncRNAs and mRNAs in the honey bee brain was performed by using Cufflinks v2.1.1 [26]. In the 1-d dinotefuran-treated group (DT_1d), compared with the 1-d untreated control group (C_1d), 90 lncRNA and 348 mRNA transcripts were upregulated, whereas 222 lncRNA and 993 mRNA transcripts were downregulated. In the 5-d dinotefuran-treated group (DT_5d), compared with the 5-d untreated control group (C_5d), 138 lncRNA and 451 mRNA transcripts were upregulated, whereas 209 lncRNA and 1007 mRNA transcripts were downregulated. In the 10-d dinotefuran-treated group (DT_10d), compared with the 10-d untreated control group (C_10d), 220 lncRNA and 608 mRNA transcripts were upregulated, whereas 125 lncRNA and 547 mRNA transcripts were downregulated (Table 1).

**Table 1:** Number of differentially expressed transcripts in each comparison

Transcripts	Regulated	DT_1d vs. C_1d	DT_5d vs. C_5d	DT_10d vs. C_10d
lncRNA	up	90	138	220
	down	222	209	125
mRNA	up	348	451	608
	down	993	1007	547

**Fig. 3:**
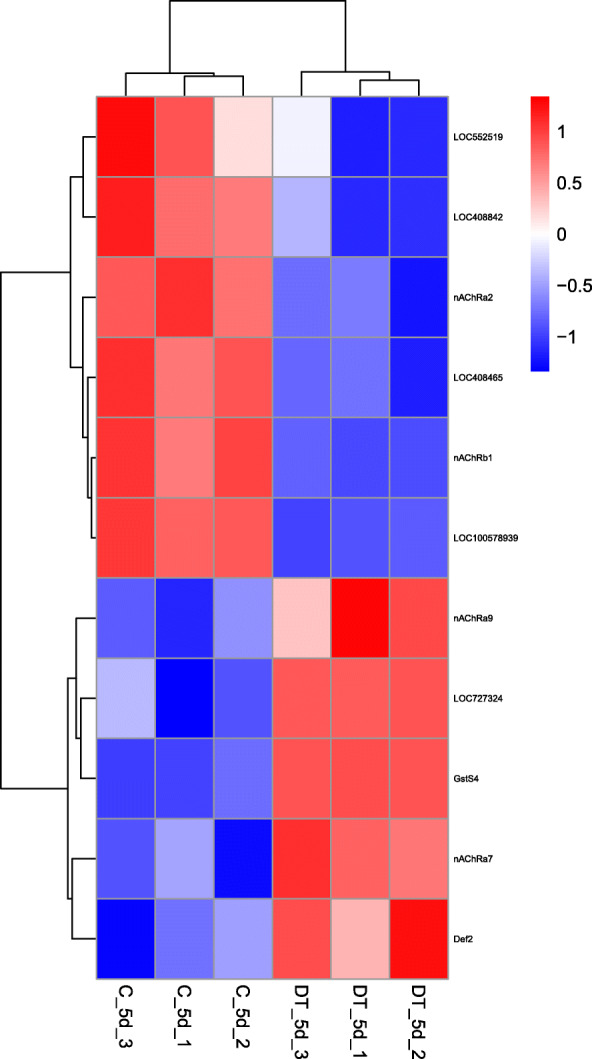
Hierarchical clustering of differentially-expressed nicotinic acetylcholine receptor and immune response-related genes. Red indicates relatively high expression, and blue indicates relatively low expression

Because dinotefuran acts on nicotinic acetylcholine receptors in honey bees and in accordance with the results of previous study, we focused on nicotinic acetylcholine receptors and immune-related genes in the GO enrichment analysis [5, 27] (Fig. 3). The expression levels of nicotinic acetylcholine receptor (nAChRb1, nAChRa9, nAChRa7 and nAChRa2) transcripts were significantly DE between dinotefuran-treated and untreated control bees at 5 d postemergence (*P* < 0.05). In addition, the expression of immune-related genes such as the single Ig IL-1-related receptor (LOC100578939), transportin-1 (LOC408842), glutathione S-transferase S4 (GstS4), prolactin-releasing peptide receptor (LOC727324), clustered mitochondria protein homolog (LOC552519), and defensin 2 (Def2), and vesicle-associated membrane protein 2 (LOC408465) genes differed significantly relative to that of control bees following dinotefuran treatment (*P* < 0.05).

### GO and KEGG analysis of target genes

**Fig. 4:**
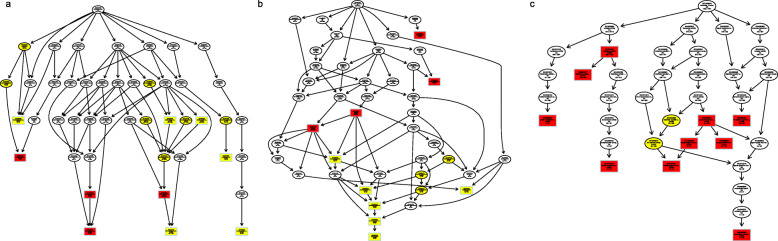
GO categorization of target genes of the DE lncRNAs identified in DT_1d vs. C_1d. (**a**) Biological processes; (**b**) cellular components; and (**c**) molecular Functions. The most significant enrichment is indicated by red, followed by yellow. Rectangles represent the top 10 GO terms of enrichment, and circles represent other GO terms

**Fig. 5:**
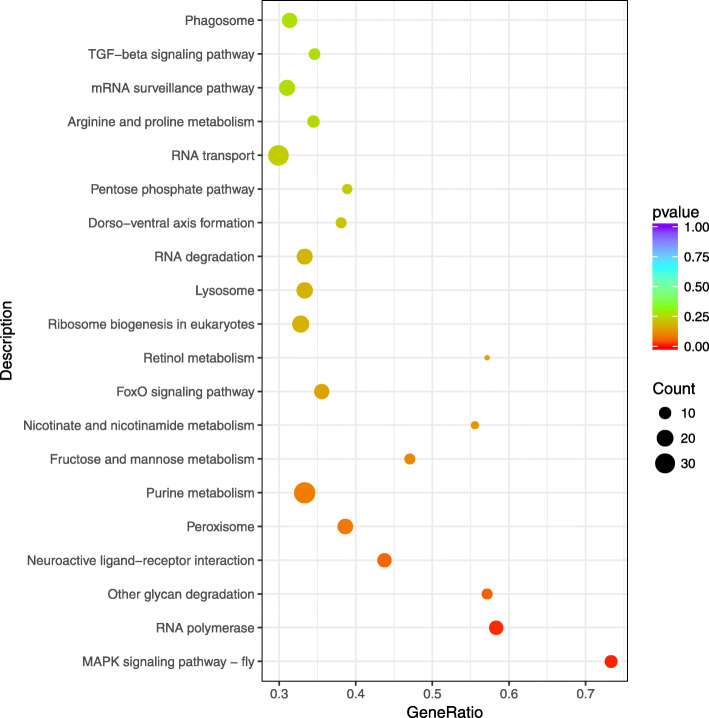
KEGG pathway enrichment analysis of the target genes of DE lncRNAs identified in DT_1d vs. C_1d

In the category of *cis* regulation, a total of 5175 target genes of DE lncRNAs were predicted among the results of the DT_1d vs. C_1d, DT_5d vs. C_5d, and DT_10d vs. C_10d comparisons. GO analysis demonstrated that the target genes of the DE lncRNAs identified in DT_1d vs. C_1d were enriched in 42 GO terms, including nucleic acid metabolic process, RNA metabolic process, hydrolase activity and RNA polymerase II transcription factor binding transcription factor activity (Fig. 4). The results of KEGG pathway enrichment analysis showed that the target genes of the DE lncRNAs identified in DT_1d vs. C_1d were enriched in terms associated with metabolism (e.g., purine metabolism, nicotinate and nicotinamide metabolism, and fructose and mannose metabolism), RNA information processing (e.g., RNA polymerase, RNA degradation, RNA transport and mRNA surveillance pathway) and signaling pathways (e.g., mitogen-activated protein kinase signaling pathway, TGF-β signaling pathway and FoxO signaling pathway) (Fig. 5). The putative target genes of the DE lncRNAs were associated mainly with neurological system processes and immune effector processes in the comparison of the 5-d and 10-d dinotefuran-treated groups (Additional file 5). In addition, the target genes were associated with RNA polymerase, purine metabolism, the MAPK signaling pathway, neuroactive ligand-receptor interaction, ECM-receptor interaction, and DNA replication in both the DT_5d vs. C_5d and DT_10d vs. C_10d comparisons (Additional file 6). Based on the *trans* regulation of lncRNAs [28], 245 target genes were predicted. We found that histone acetylation, protein acetylation and amino sugar metabolic processes were the most common significantly enriched terms in the three comparison groups (*P* < 0.05) (Additional file 7). Additionally, ribosome biogenesis in eukaryotes and DNA replication were the most enriched pathways in each of the comparisons (Additional file 8).

### GSEA of mRNA transcripts

**Fig. 6:**
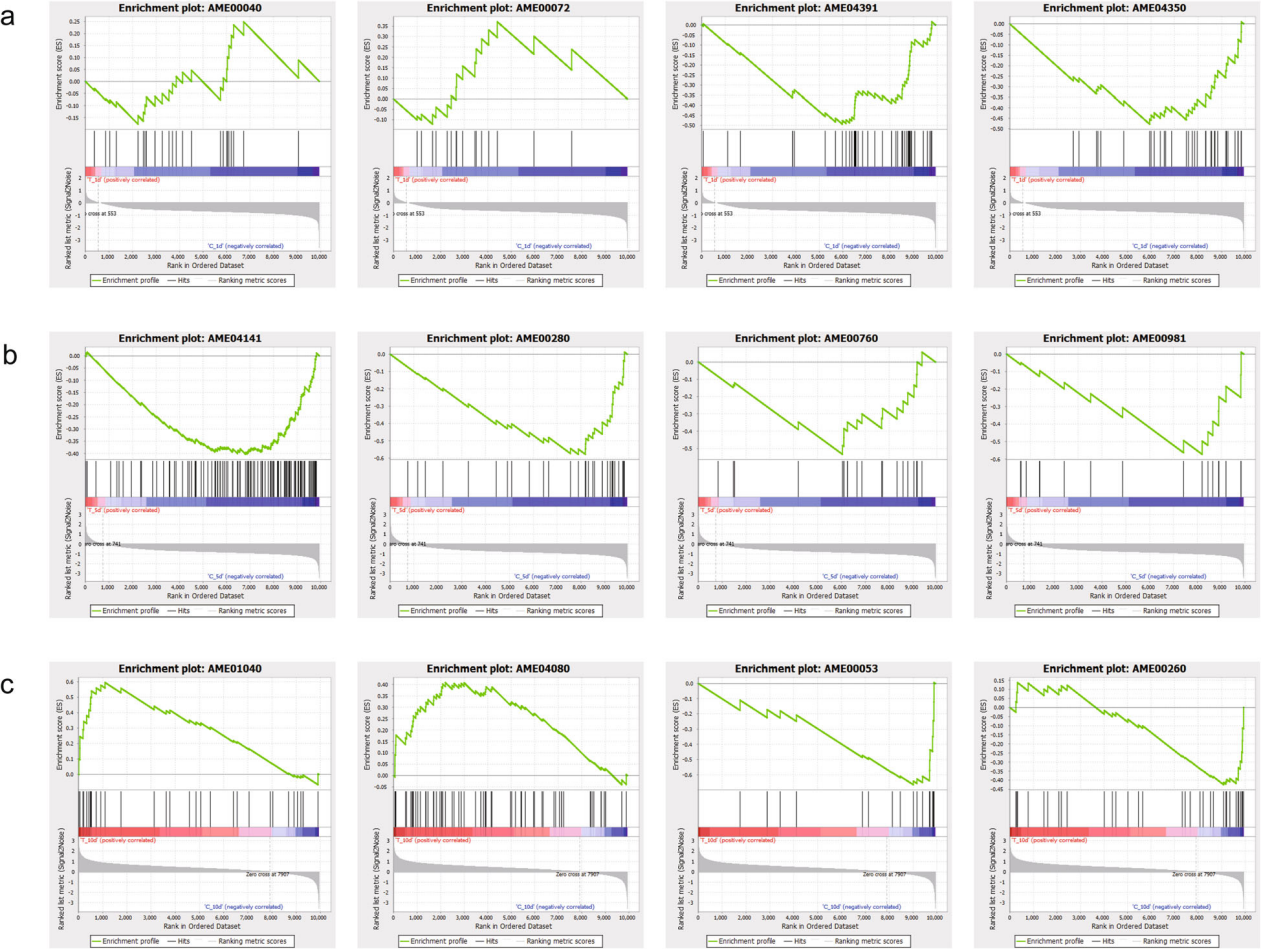
GSEA of mRNA expression levels in the honey bee brain. (**a**) The enriched pathways of 1-d dinotefuran-treated group; (**b**) the enriched pathways of 5-d dinotefuran-treated group; (**c**) the enriched pathways of 10-d dinotefuran-treated group

To avoid missing valuable information from genes showing nonsignificantly DE but biologically important, GSEA was applied for gene enrichment analysis [29] (Fig. 6 and Additional file 9). The 1-d dinotefuran-treated group was significantly associated with the synthesis and degradation of ketone bodies (AME00072, NES = 1.748, *P* = 0.012), pentose and glucuronate interconversions (AME00040, NES = 1.554, *P* = 0.027), the Hippo signaling pathway (AME04391, NES = -2.138, *P* < 0.001) and the TGF-β signaling pathway-fly (AME04350, NES = -1.936, *P* < 0.001) (Fig. 6a). The enriched gene pathways of the 5-d dinotefuran-treated group were related to protein processing in the endoplasmic reticulum (AME04141, NES = -1.890, *P* < 0.001), valine, leucine and isoleucine degradation (AME00280, NES = -2.256, *P* < 0.001), nicotinate and nicotinamide metabolism (AME00760, NES = -1.833, *P* = 0.002) and insect hormone biosynthesis (AME00981, NES = 1.894, *P* = 0.003) (Fig. 6b). The 10-d dinotefuran-treated group exhibited a tendency for high enrichment in pathways such as the biosynthesis of unsaturated fatty acids (AME01040, NES = 1.835, *P* < 0.001), neuroactive ligand-receptor interaction (AME04080, NES = 1.372, *P* = 0.026), ascorbate and aldarate metabolism (AME00053, NES = -2.567, *P* < 0.001), and glycine, serine and threonine metabolism (AME00260,NES = -1.892, *P* < 0.001) (Fig. 6c). Interestingly, transcripts related to glycine, serine and threonine metabolism, and pentose and glucuronate interconversion were found to be enriched after 1, 5, and10 d of repeated dietary exposure to dinotefuran daily, suggesting an effect on the metabolism of carbohydrates and proteins in honey bees.

### Subcellular localization of lncRNA TCONS_00086519

**Fig. 7:**
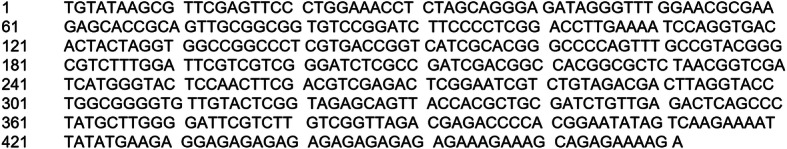
Schematic diagram of the full-length 471 bp honey bee brain lncRNA TCONS_0008651 sequence

**Fig. 8:**
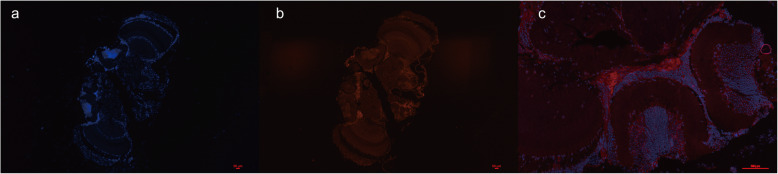
The location of RNA TCONS_00086519 in the honey bee brain. (**a**) RNA fluorescence in situ hybridization (FISH) signal location using probes against TCONS_00086519; (**b**) the nucleus was stained with DAPI; (**c**) lncRNA TCONS_00086519 is distributed predominantly in the cytoplasmic region in the honey bee brain

To explore the potential function of lncRNAs in the honey bee brain, the differentially and highly expressed lncRNA TCONS_00086519, which showed a full-length sequence of 471 bp (Fig. 7), was selected for the next study. Four protein-coding genes were found close to TCONS_0008615 through bioinformatic analysis (Additional file 10). The subcellular localization of lncRNAs is closely associated with their biological mechanism [30]. To further investigate the precise mechanism of the lncRNAs, an RNA-FISH assay was performed to detect the subcellular localization of TCONS_00086519. As shown in Fig. 8, TCONS_00086519 was mainly distributed in the cytoplasm in honey bee brain cells. This finding provided evidence that TCONS_00086519 may act as a competing endogenous RNA (ceRNA) or a molecular sponge to modulate the expression of its target mRNA or miRNA.

### Quantitative real-PCR validation

**Fig. 9:**
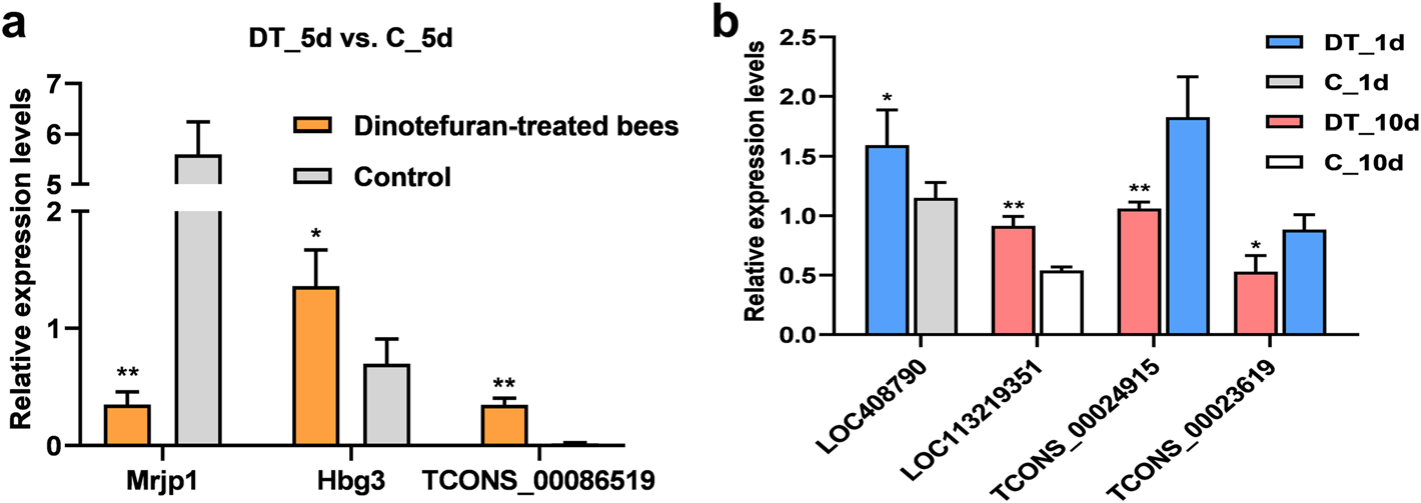
Validation of RNA-seq data by qRT–PCR. (**a**) Relative expression levels of differentially expressed transcripts between dinotefuran-treated bees and control bees at 5 d; (**b**) relative expression levels of differentially expressed transcripts identified in each of the DT_1d vs. C_1d, DT_10d vs. C_10d and DT_10d vs. DT_1d comparisons. The expression data are presented as the mean ± SEM. *P < 0.05, **P < 0.01

To validate the RNA-seq results, four DE mRNAs (Mrjp1, LOC408790, LOC113219351 and Hbg3) and three DE lncRNAs (TCONS_00086519, TCONS_00024915 and TCONS_00023619) were subjected to qPCR. As shown in Fig. 9, Hbg3, TCONS_00086519, LOC408790 and LOC113219351 exhibited significantly higher expression in dinotefuran-treated bees than in control bees (*P* < 0.05), which was consistent with the RNA-seq data. The expression levels of TCONS_00024915 and TCONS_00023619 decreased significantly after persistent exposure to dinotefuran (*P* < 0.05). Moreover, Mrjp1 expression showed a 14.9-fold reduction relative to controls after dinotefuran treatment for 5 d.

## Discussion

Studies on lncRNA expression in honey bees have been increasing in recent years, focused mainly on caste determination [19, 31], the behavioral transition from nurse to forager [25], oviposition [32, 33], and the response to pathogen infestation [20, 21, 34]. In the current study, RNA-seq was performed to identify the lncRNA and mRNA profiles of honey bees exposed to 0.01 mg/L dinotefuran for 1, 5, and 10 d to assess the potential regulators of honey bee growth and immune responses related to neonicotinoid insecticides. Finally, 6824 putative lncRNA transcripts and 19,837 known mRNA transcripts were obtained in the honey bee brain transcriptome. A total of 312 lncRNAs and 1341 mRNAs, 347 lncRNAs and 1458 mRNAs, 345 lncRNAs and 1155 mRNAs were identified as DE in the DT_1d vs. C_1d, DT_5d vs. C_5d, and DT_10d vs. C_10d comparisons, respectively. Among these transcripts, we found that several DE lncRNAs were actively associated with honey bee growth and immune responses after dinotefuran treatment. The lncRNA TCONS_00064387 exhibited lower expression in the dinotefuran-treated group than in the control group and targeted the Mrjp family genes in *cis*. Mrjp family genes have been validated and determined in honey bees, and encode major royal jelly proteins which are the major organic components of royal jelly [35]. The royal jelly master proteins Mrjp1-Mrjp8 have been repeatedly reported in the brain of honey bees [36–38] and play crucial neurobiological roles in the nervous system [39].The expression of Mrjp1 was detected in the mushroom body [36], optic lobe [38] and antennal lobe [40], and reduced expression of the Mrjp1 gene in the mushroom body decreased learning ability in worker honey bees [41]. The effect of sublethal concentrations of dinotefuran on reducing the expression of Mrjps suggested that the learning ability and nervous system of honey bees may be affected. The nAChRs are the targets of neonicotinoid insecticides [2], and five nAChR subunits (nAChRα4, the putative target of TCONS_00040953; nAChRα7, the target of XR_001705519.2; nAChRα8, the target of TCONS_00031755, TCONS_00031757 and TCONS_00031758; nAChRα9, the target of XR_003306308.1 and XR_003304779.1; and nAChRβ4, the target of XR_001705519.2) were separately investigated for their effects in *trans*. The induction of nAChRs can result in distinct effects; for instance, exposure to sublethal dinotefuran doses can affect olfaction, basic motor function and postural control in honey bees [12, 42, 43]. Defensin genes, which play an important role in the innate humoral immune system of honey bees and are associated with responses against G- and, G+ bacteria and fungi, showed significantly lower expression in the dinotefuran-treated group [44, 45]. Defensin1 and Defensin 2 were identified as candidate targets of the annotated DE lncRNAs XR_003304788.1 and XR_003306226.1, respectively. This result is consistent with a previous study that showed that thiamethoxam, another neonicotinoid insecticide, was able to suppress defensin gene expression [46]. In general, our results may provide insights into the effect of dinotefuran on the immune system of *A. mellifera* from the perspective of lncRNAs. These findings suggest that DE lncRNAs may participate in the response to dinotefuran through *cis* and *trans* effects.

Through GSEA with mRNA expression data sets and the target genes identified from DE lncRNA enrichment analysis, we found that the TGF-β signaling pathway was the most enriched pathway in both mRNAs and the target genes of lncRNAs. This result is in agreement with another neonicotinoid insecticide on adult honey bees that were exposed to realistic field doses of clothianidin [47]. A previous study showed that *Varroa destructor* activated the TGF-β signaling pathway to suppress wound healing and the immune response in honey bees [48]. Similarly, TGF-β signaling interferes with innate immunity in *Drosophila* larvae infected with symbiotic nematodes, and phenoloxidase activity in the hemolymph of infected larvae is regulated by activin signaling via TGF-β pathways [49]. This suggests that transforming growth factor-β (TGF-β) may play a critical role in the honey bee immune response to dinotefuran. In this study, mitogen-activated protein kinase 1 (LOC409523), which was enriched in the TGF-β signaling pathway, was indicated to be a potential target of DE lncRNA TCONS_00018497 and showed significantly lower expression in dinotefuran-treated bees. MAPK was associated with decreased developmental time and increased titer of juvenile hormone (JH) [50], a hormone that is essential for development and affects the structure and function of the adult honey bee nervous system [51, 52]. The MAPK pathway has been reported to be involved in the antiviral immune responses of honey bees [53]. Moreover, pathway analyses demonstrated that a variety of mRNA expression data sets and target genes of DE lncRNAs in both comparison groups were enriched in material and energy metabolism-associated pathways, such as glycine, serine and threonine metabolism, and pentose and glucuronate interconversion. Metabolism plays an important role in the regulation of sustenance to innate and adaptive immune responses [54]. These results indicated that DE lncRNAs may act as regulators in the development, nerve conduction, and immune response of honey bees treated with dinotefuran.

Accumulating evidence indicates that lncRNAs participate in gene expression regulation by modulating chromosomal architecture [55, 56], binding to transcription factors for direct recruitment [57], and RNA polymerase II [58] in the nucleus. However, several lncRNAs were found to control mRNA stability and modulate translation and posttranslational modifications in the cytoplasm [14, 59, 60]. Consequently, investigation into the subcellular localization of lncRNAs will be helpful to further illustrate the mechanisms and functions of lncRNAs [14, 61]. Through RNA sequencing, we obtained the highly and significantly DE lncRNA TCONS_00086519, with a length of 471 bp, which potentially targets four genes in *trans*, including general odorant-binding protein 71 and smoothelin-like protein 1. The results indicated that TCONS_00086519 may be associated with odorant binding, development and immunity [62]. RNA FISH analysis revealed that TCONS_00086519 was mainly located in the cytoplasm, suggesting that it is capable of forming complexes with diverse structural and regulatory functions related to mRNA stability, mRNA translation, and signaling pathway modulation, serving as a competing endogenous RNA, or functioning as a precursor of microRNAs [60, 63]. The results of this work provide a foundation for further study of the function and mechanism of lncRNA TCONS_00086519 in the honey bee brain.

## Conclusion

This study revealed the expression patterns of lncRNAs in honey bee brains following exposure to a 0.01 mg/L dose of dinotefuran. Bioinformatic analysis showed that several lncRNAs and mRNAs were likely to participate in important biological processes associated with honey bee material and energy metabolism, neuroactive ligand-receptor interaction and odorant binding. Additionally, we found that the DE lncRNA TCONS_00086519 was distributed mainly in the cytoplasm and may act as a regulator by serving as a miRNA precursor or ceRNA involved in development and the immune response by regulating gene expression in *trans* in honey bees. Our results provide a foundation for understanding the lncRNA-mediated regulation of growth and immunity and contribute to a better understanding of honey bee-insecticide interactions and honey bees themselves.

## Methods and materials

### Honey bee rearing

Frames containing sealed broods (near adult emergence) were collected from three healthy colonies located at the Zhejiang Academy of Agricultural Sciences (Hangzhou, China) and maintained in darkness in a climate-controlled incubator (34 °C ± 1 °C, relative humidity [RH] 60 ± 10%). Then, we obtained newly emerged honey bees and placed them in cages (11 × 11 × 7 cm^3^, *N* = 60 in each cage).

### Dinotefuran preparation and exposure

The residues of dinotefuran recorded in pollen, honey or syrup range from 0.03 ng/g to 147 ng/g [11, 64–66]. On this basis, a field-realistic level of dinotefuran of 0.01 mg/L was selected as the sublethal concentration in this study. A dinotefuran (> 99% purity, Sigma-Aldrich, St Louis, MO, USA) stock solution (1000 ng a.i./L) was prepared using a 50% sucrose:water solution as the solvent. The bees were treated with 2 mL of 0.01 mg/L dinotefuran solution in the experimental group (DT). Fifteen honey bees were collected on the 1st day (DT_1d), 5th day (DT_5d) and 10th day (DT_10d). The bees in the control group were fed 2 mL of a 50% sucrose: water solution, and samples were collected at the same time on the 1st day (C_1d), 5th day (C_5d) and 10th day (C_10d). The honey bees were fed ad libitum. Dead bees were removed and both treatment solution and untreated sucrose solution were replaced daily throughout the experiment. The bee samples were preserved in liquid nitrogen and stored at − 80 °C until brain dissection. The in vivo portion of the study was carried out at 34 °C ± 1 °C under 60 ± 10% RH in darkness.

### Library preparation and sequencing

Total RNA was isolated from 15 pooled brains per sample using TRIzol reagent (Invitrogen, Carlsbad, CA, USA). RNA was checked for purity and integrity by using a NanoPhotometer® spectrophotometer (IMPLEN, CA, USA) and a Bioanalyzer 2100 system (Agilent Technologies, CA, USA). A total of 3 μg RNA per sample was used to generate a complementary library with the NEBNext® Ultra™ RNA Library Prep Kit for Illumina® (NEB, Ipswich, MA, USA) following the manufacturer’s recommendations. After cluster generation using the TruSeq PE Cluster Kit v3-cBot-HS (Illumina, USA) according to the manufacturer’s instructions, 18 libraries were sequenced on the Illumina HiSeq 2500 platform, and 150 bp paired-end reads were generated.

### Identification of lncRNA and novel mRNA

Clean data were obtained after discarding the reads containing adapters, reads containing poly-Ns sequences (over 0.2%) and reads of low quality (over 50%) from the raw data. Q_20_ and Q_30_ values and GC contents were calculated to evaluate sequencing quality. The remaining clean data were aligned to the reference *A. mellifera* genome (https://www.ncbi.nlm.nih.gov/assembly/GCF_003254395.2) with Hisat2 (https://ccb.jhu.edu/software/hisat2/index.shtml) [67]. All mapped reads were assembled by StringTie [68] using the default parameters. The transcripts obtained from splicing were merged by using Cuffmerge, and transcripts of less than 200 nt in length with an uncertain strand orientation were removed. The obtained sequences were subjected to BlAST searches against the reference database, and known transcripts were filtered out by Cuffcompare [69]. The remaining transcripts were considered candidate lncRNAs, and those with coding potential were considered novel mRNAs.

### Differential expression analysis

The expression levels of mRNAs and lncRNAs were calculated as fragments per kilobase of exon per million mapped fragments (FPKM) values using the Cuffdiff tool in Cufflinks v2.1.1 [26]. A *P*-value < 0.05 (Benjamini and Hochberg’s false discovery rate) and a |log_2_ (fold change) | > 1 were set as the thresholds for significantly differential expression.

### RNA fluorescence in situ hybridization (RNA FISH)

The specific Cy3-labeled oligonucleotide probe TCONS_00086519: 5′-Cy3-CCGAGTCTCG.

ACGTCGAAGTTGGAGTACCCATGATCGACCGTTAG-3′ was designed and synthesized. RNA FISH was performed using an RNA FISH Kit (Gefanbio, Shanghai, China) according to the manufacturer’s instructions. Briefly, honey bee brains were dissected and fixed with RNA-free paraformaldehyde. The paraffin sections were pretreated for hybridization with a 30% H_2_O_2_-methanol mixture (1:9) for 10 min at room temperature (RT). Then, the sections were treated via proteinase K digestion (20 min, 37 °C) in 25% HCl, followed by a 1-min wash in 0.2% glycine irrigation solution. After fixation in 4% formaldehyde for 10 min, the sections were washed twice in acetic anhydride (pH = 8.0) at RT for 5 min each time. The sections were incubated in a humidified chamber (1 h, 65 °C) covered with the prehybridization solution. The tissue sections were hybridized with the probe at 0.1 μM for 48 h at 65 °C. The tissues were washed twice on coverslips with 2 × SSC (pH = 7.5). Finally, the sections were stained with DAPI, embedded in mounting agent, and then observed under a fluorescence microscope (IX-71, Olympus, Japan).

### Target gene prediction

Differentially expressed lncRNAs were selected to predict potential *cis* and *trans* effects. The *cis* actions of lncRNAs affect neighboring target genes. Based on the positional relationship between lncRNAs and mRNAs, target genes were predicted in the regions 100 kb up- and downstream of the DE lncRNAs. Based on the expression correlation between lncRNAs and mRNAs, the target genes were evaluated for a *trans* effect of lncRNAs with an absolute Pearson correlation ≥0.95.

### Enrichment analysis

Gene Ontology (GO) enrichment analysis of the target genes of identified DE lncRNAs was performed using the GOseq R package [70], and Kyoto Encyclopedia of Genes and Genomes (KEGG, www.kegg.jp/kegg/kegg1.html) pathway analysis of the target genes was implemented by using KOBased Annotation System (KOBAS) v2.0 [71], considering a corrected *p*-value < 0.05 to indicate significant enrichment. GSEA software (http://software.broadinstitute.org/gsea/index.jsp) was applied to interpret gene expression data [29, 72]. Briefly, the genes were metrically ranked according to the signal-to-noise ratio ($\frac{\upmu \mathrm{a}-\upmu \mathrm{b}}{\upsigma \mathrm{a}+\upsigma \mathrm{b}}$). Then, it was determined whether the genes in the gene set database (KEGG and GO databases) were ranked at the top or bottom of the list in terms of enrichment. The significance of the enrichment score (ES) was calculated with an empirical phenotypic-based permutation test. The normalized enrichment scores (NES) were used to compare the analysis results across gene sets, accounting for differences in gene set size and in the correlations between the gene sets and the expression dataset. The false discovery rate was calculated to control the false positive rate. IN GSEA, NES are determined as follows:

$$ \mathrm{NES}=\frac{actual\  ES}{\  mean\Big( ESs\  against\  all\  permutations\ of\ the\ dataset} $$

### Quantitative PCR analysis

First strand cDNA was obtained with the PrimeScript™ RT reagent Kit with gDNA Eraser (TaKaRa, Dalian, China). Quantitative real-time PCR was performed on an ABI 7500 Real-Time PCR system (Applied Biosystem, Carlsbad, CA, USA) using TB Green® *Premix Ex Taq*™ II (TaKaRa, Dalian, China) with specific primers (Table 2). Relative gene expression levels were quantified based on β-actin expression by using the 2^-∆∆Ct^ method with three independent biological replicates. The differences in the relative expression levels of the mRNAs and lncRNAs identified between the dinotefuran-treated and control bees were analyzed using one-way ANOVA and an independent-samples t-test with SPSS 22.0 software (IBM, Armonk, NY, USA). *p* < 0.05 was considered statistically significant.

**Table 2:** The sequences of primers for the selected lncRNAs and mRNAs

Primer Name	Sequences (5′-3′)
β-actin-F	TGCCAACACTGTCCTTTCTG
β-actin-R	AGAATTGACCCACCAATCCA
LOC408790-F	TTGACTCTATCTCCCCGGCA
LOC408790-R	TCTGCTTTCAAAATGCGCGTAA
LOC113219351-F	GCTGCGGATATGGGTACGAA
LOC113219351-R	GTTTGGAACGCGAAGAGCAC
Hbg3-F	AAGCCGCTCCCTGAAAACTT
Hbg3-R	ATGTCGACCCCCATTTCGAG
TCONS_00086519-F	TCTTTGGATTCGTCGTCGGG
TCONS_00086519--R	TATTCCGTGGGGTCTCGTCT
TCONS_00024915-F	AGTGATGGCACAGCTGAACA
TCONS_00024915-R	GACGTGAAGGACTCAACCGT
TCONS_00033704-F	CCGTGGAGTTAGGGTGAGTC
TCONS_00033704-R	ACAATACTTGCAGCCATTAGCA

## Supplementary Information

**Additional file 1.**

**Additional file 2.**

**Additional file 3.**

**Additional file 4.**

**Additional file 5.**

**Additional file 6.**

**Additional file 7.**

**Additional file 8.**

**Additional file 9.**

**Additional file 10.**

## Data Availability

The sequencing data are available in the GEO database (GSE168740) of the NCBI system (https://www.ncbi.nlm.nih.gov/geo/query/acc.cgi?acc=GSE168740).

## Abbreviations

CNCI: Coding-Non-Coding Index
CPC: Coding Potential Calculator
CSP: chemosensory proteins
FISH: fluorescence in situ hybridization
GSEA: Gene set enrichment analysis
GstS4: glutathione S-transferase S4
JH: juvenile hormone
MAPK: Mitogen-activated protein kinase
Mrjp: major royal jelly protein family
nAChR: nicotinic acetylcholine receptor
ND1: NADH dehydrogenase subunit 1
ORFs: open reading frames
TGF-β: transforming growth factor-β

## References

[1] Bass C, Denholm I, Williamson MS, Nauen R (2015). The global status of insect resistance to neonicotinoid insecticides. Pestic Biochem Physiol.

[2] Jeschke P, Nauen R (2008). Neonicotinoids—from zero to hero in insecticide chemistry. Pest Manag Sci.

[3] Goulson D (2013). REVIEW: an overview of the environmental risks posed by neonicotinoid insecticides. J Appl Ecol.

[4] Peng Y, Yang E (2016). Sublethal dosage of Imidacloprid reduces the microglomerular density of honey bee mushroom bodies. Sci Rep.

[5] Li Z, Yu T, Chen Y, Heerman M, He J, Huang J, Nie H, Su S (2019). Brain transcriptome of honey bees (Apis mellifera) exhibiting impaired olfactory learning induced by a sublethal dose of imidacloprid. Pestic Biochem Physiol.

[6] Mengoni Goñalons C, Farina WM (2015). Effects of sublethal doses of Imidacloprid on Young adult honeybee behaviour. PLoS One.

[7] Henry M. A common pesticide decreases foraging success and survival in honey bees. Sci. 2012. 10.1126/science.1215039 22461498

[8] Tackenberg MC, Giannoni-Guzmán MA, Sanchez-Perez E, Doll CA, Agosto-Rivera JL, Broadie K, Moore D, McMahon DG (2020). Neonicotinoids disrupt circadian rhythms and sleep in honey bees. Sci Rep.

[9] Wakita T, Kinoshita K, Yamada E, Yasui N, Kawahara N, Naoi A, Nakaya M, Ebihara K, Matsuno H, Kodaka K (2003). The discovery of dinotefuran: a novel neonicotinoid. Pest Manag Sci.

[10] The Pesticide Properties Database PPDB [http://sitem.herts.ac.uk/aeru/iupac/index.htm].

[11] Dively GP, Kamel A (2012). Insecticide residues in pollen and nectar of a cucurbit crop and their potential exposure to pollinators. J Agric Food Chem.

[12] Liu S, Liu Y, He F, Zhang H, Li X, Tan H (2019). Enantioselective olfactory effects of the neonicotinoid Dinotefuran on honey bees (Apis mellifera L.). J Agric Food Chem.

[13] Matsumoto T (2013). Reduction in homing flights in the honey bee Apis mellifera after a sublethal dose of neonicotinoid insecticides. Bull Insectol.

[14] Fatica A, Bozzoni I (2014). Long non-coding RNAs: new players in cell differentiation and development. Nat Rev Genet.

[15] Fernandes J, Acuña S, Aoki J, Floeter-Winter L, Muxel S. Long Non-Coding RNAs in the Regulation of Gene Expression: Physiology and Disease. Non-Coding RNA. 2019;5(1):17. 10.3390/ncrna5010017. 10.3390/ncrna5010017 PMC6468922 30781588

[16] Hadjicharalambous MR, Lindsay MA (2019). Long non-coding RNAs and the innate immune response. Non-Coding RNA.

[17] Honson DD, Macfarlan TS (2018). A lncRNA-like role for LINE1s in development. Dev Cell.

[18] Tadano H, Yamazaki Y, Takeuchi H, Kubo T (2009). Age- and division-of-labour-dependent differential expression of a novel non-coding RNA, Nb-1, in the brain of worker honeybees, Apis mellifera L. Insect Mol Biol.

[19] Humann FC, Tiberio GJ, Hartfelder K (2013). Sequence and expression characteristics of long noncoding RNAs in honey bee caste development – potential novel regulators for transgressive ovary size. PLoS One.

[20] Jayakodi M, Jung JW, Park D, Ahn Y-J, Lee S-C, Shin S-Y, Shin C, Yang T-J, Kwon HW (2015). Genome-wide characterization of long intergenic non-coding RNAs (lincRNAs) provides new insight into viral diseases in honey bees Apis cerana and Apis mellifera. BMC Genomics.

[21] Chen D, Chen H, Du Y, Zhou D, Geng S, Wang H, Wan J, Xiong C, Zheng Y, Guo R (2019). Genome-wide identification of long non-coding RNAs and their regulatory networks involved in Apis mellifera ligustica response to Nosema ceranae infection. Insects.

[22] Guo R, Chen D, Xiong C, Hou C, Zheng Y, Fu Z, Diao Q, Zhang L, Wang H, Hou Z, Li W, Kumar D, Liang Q (2018). Identification of long non-coding RNAs in the chalkbrood disease pathogen Ascospheara apis. J Invertebr Pathol.

[23] Satyavathi V, Ghosh R, Subramanian S (2017). Long non-coding RNAs regulating immunity in insects. Non-Coding RNA.

[24] Shen Y, Mao H, Huang M, Chen L, Chen J, Cai Z, Wang Y, Xu N (2016). Long noncoding RNA and mRNA expression profiles in the thyroid gland of two phenotypically extreme pig breeds using Ribo-zero RNA sequencing. Genes.

[25] Liu F, Shi T, Qi L, Su X, Wang D, Dong J, Huang ZY: lncRNA profile of Apis mellifera and its possible role in behavioural transition from nurses to foragers. BMC Genomics 2019, 20(1):393, DOI: 10.1186/s12864-019-5664-7. 10.1186/s12864-019-5664-7 PMC6528240 31113365

[26] Trapnell C, Williams BA, Pertea G, Mortazavi A, Kwan G, van Baren MJ, Salzberg SL, Wold BJ, Pachter L (2010). Transcript assembly and quantification by RNA-Seq reveals unannotated transcripts and isoform switching during cell differentiation. Nat Biotechnol.

[27] Li Z, Li M, He J, Zhao X, Chaimanee V, Huang W, Nie H, Zhao Y, Su S (2017). Differential physiological effects of neonicotinoid insecticides on honey bees: a comparison between Apis mellifera and Apis cerana. Pestic Biochem Physiol.

[28] Shu X, Shu S, Cheng H (2019). A novel lncRNA-mediated trans-regulatory mechanism in the development of cleft palate in mouse. Mol Genet Genomic Med.

[29] Subramanian A, Tamayo P, Mootha VK, Mukherjee S, Ebert BL, Gillette MA, Paulovich A, Pomeroy SL, Golub TR, Lander ES, Mesirov JP (2005). Gene set enrichment analysis: a knowledge-based approach for interpreting genome-wide expression profiles. Proc Natl Acad Sci U S A.

[30] Kopp F, Mendell JT (2018). Functional classification and experimental dissection of long noncoding RNAs. Cell.

[31] Collins DH, Wirén A, Labédan M, Smith M, Prince DC, Mohorianu I, Dalmay T, Bourke AFG (2021). Gene expression during larval caste determination and differentiation in intermediately eusocial bumblebees, and a comparative analysis with advanced eusocial honeybees. Mol Ecol.

[32] Chen X, Ma C, Chen C, Lu Q, Shi W, Liu Z, Wang H, Guo H (2017). Integration of lncRNA-miRNA-mRNA reveals novel insights into oviposition regulation in honey bees. PeerJ.

[33] Chen X, Shi W (2020). Genome-wide characterization of coding and non-coding RNAs in the ovary of honeybee workers and queens. Apidologie.

[34] Lin Z, Liu Y, Chen X, Han C, Wang W, Ke Y, Su X, Li Y, Chen H, Xu H, Chen G, Ji T (2020). Genome-wide identification of long non-coding RNAs in the gravid Ectoparasite Varroa destructor. Front Genet.

[35] Schmitzová J, Klaudiny J, Albert S, Schröder W, Schreckengost W, Hanes J, Júdová J, Simúth J (1998). A family of major royal jelly proteins of the honeybee Apis mellifera L. Cell Mol Life Sci.

[36] Kucharski R, Maleszka R, Hayward DC, Ball EE (1998). A royal jelly protein is expressed in a subset of Kenyon cells in the mushroom bodies of the honey bee brain. Naturwissenschaften.

[37] Whitfield CW, Band MR, Bonaldo MF, Kumar CG, Liu L, Pardinas JR, Robertson HM, Soares MB, Robinson GE (2002). Annotated expressed sequence tags and cDNA microarrays for studies of brain and behavior in the honey bee. Genome Res.

[38] Peixoto LG, Calábria LK, Garcia L, Capparelli FE, Goulart LR, de Sousa MV, Espindola FS (2009). Identification of major royal jelly proteins in the brain of the honeybee Apis mellifera. J Insect Physiol.

[39] Zhang X, Hu H, Han B, Wei Q, Meng L, Wu F, Fang Y, Feng M, Ma C, Rueppell O, Li J (2020). The Neuroproteomic basis of enhanced perception and processing of brood signals that trigger increased reproductive Investment in Honeybee (Apis mellifera) workers. Mol Cell Proteomics.

[40] Rosmilah M, Shahnaz M, Patel G, Lock J, Rahman D, Masita A, Noormalin A (2008). Characterization of major allergens of royal jelly Apis mellifera. Trop Biomed.

[41] Hojo M, Kagami T, Sasaki T, Nakamura J, Sasaki M (2010). Reduced expression of major royal jelly protein 1 gene in the mushroom bodies of worker honeybees with reduced learning ability. Apidologie.

[42] Williamson SM, Willis SJ, Wright GA (2014). Exposure to neonicotinoids influences the motor function of adult worker honeybees. Ecotoxicology.

[43] Alkassab AT, Kirchner WH (2017). Sublethal exposure to neonicotinoids and related side effects on insect pollinators: honeybees, bumblebees, and solitary bees. J Plant Dis Prot.

[44] Richard F-J, Holt HL, Grozinger CM (2012). Effects of immunostimulation on social behavior, chemical communication and genome-wide gene expression in honey bee workers (Apis mellifera). BMC Genomics.

[45] López-Uribe MM, Fitzgerald A, Simone-Finstrom M (2017). Inducible versus constitutive social immunity: examining effects of colony infection on glucose oxidase and defensin-1 production in honeybees. R Soc Open Sci.

[46] Shi TF, Wang YF, Liu F, Qi L, Yu LS. Influence of the Neonicotinoid Insecticide Thiamethoxam on miRNA Expression in the Honey Bee (Hymenoptera: Apidae). J Insect Sci. 2017;17(5):96. 10.1093/jisesa/iex074. 10.1093/jisesa/iex074 PMC7206646 29117371

[47] Morfin N, Goodwin PH, Guzman-Novoa E (2020). Interaction of field realistic doses of clothianidin and Varroa destructor parasitism on adult honey bee (*Apis mellifera* L.) health and neural gene expression, and antagonistic effects on differentially expressed genes. PLoS One.

[48] Erban T, Sopko B, Kadlikova K, Talacko P, Harant K (2019). Varroa destructor parasitism has a greater effect on proteome changes than the deformed wing virus and activates TGF-β signaling pathways. Sci Rep.

[49] Ozakman Y, Eleftherianos I. TGF-β Signaling Interferes With the Drosophila Innate Immune and Metabolic Response to Parasitic Nematode Infection. Front Physiol. 2019;10:716. 10.3389/fphys.2019.00716. 10.3389/fphys.2019.00716 PMC6611403 31316388

[50] Kamakura M (2011). Royalactin induces queen differentiation in honeybees. Nature.

[51] Scholl C, Wang Y, Krischke M, Mueller MJ, Amdam GV, Rössler W (2014). Light exposure leads to reorganization of microglomeruli in the mushroom bodies and influences juvenile hormone levels in the honeybee. Dev Neurobiol.

[52] Fahrbach SE, Robinson GE (1996). Juvenile hormone, behavioral maturation, and brain structure in the honey bee. Dev Neurosci.

[53] Brutscher LM, Daughenbaugh KF, Flenniken ML (2015). Antiviral defense mechanisms in honey bees. Curr Opin Insect Sci.

[54] Ganeshan K, Chawla A (2014). Metabolic regulation of immune responses. Annu Rev Immunol.

[55] Creamer KM, Lawrence JB. XIST RNA: a window into the broader role of RNA in nuclear chromosome architecture. Philos Trans R Soc Lond Ser B Biol Sci. 2017;372(1733):20160360. 10.1098/rstb.2016.0360. 10.1098/rstb.2016.0360 PMC5627162 28947659

[56] Hall LL, Carone DM, Gomez AV, Kolpa HJ, Byron M, Mehta N, Fackelmayer FO, Lawrence JB (2014). Stable C0T-1 repeat RNA is abundant and is associated with euchromatic interphase chromosomes. Cell.

[57] Azofeifa JG, Allen MA, Hendrix JR, Read T, Rubin JD, Dowell RD (2018). Enhancer RNA profiling predicts transcription factor activity. Genome Res.

[58] Espinoza CA, Allen TA, Hieb AR, Kugel JF, Goodrich JA (2004). B2 RNA binds directly to RNA polymerase II to repress transcript synthesis. Nat Struct Mol Biol.

[59] Zhang B, Gunawardane L, Niazi F, Jahanbani F, Chen X, Valadkhan S (2014). A novel RNA motif mediates the strict nuclear localization of a long noncoding RNA. Mol Cell Biol.

[60] Rashid F, Shah A, Shan G (2016). Long non-coding RNAs in the cytoplasm. Genomics Proteomics Bioinformatics.

[61] Das S, Zhang E, Senapati P, Amaram V, Reddy MA, Stapleton K, Leung A, Lanting L, Wang M, Chen Z, Kato M, Oh HJ, Guo Q, Zhang X, Zhang B, Zhang H, Zhao Q, Wang W, Wu Y, Natarajan R (2018). A novel angiotensin II–induced long noncoding RNA giver regulates oxidative stress, inflammation, and proliferation in vascular smooth muscle cells. Circul Res.

[62] Du L, Liu Q, Shen F, Fan Z, Hou R, Yue B, Zhang X (2019). Transcriptome analysis reveals immune-related gene expression changes with age in giant panda (Ailuropoda melanoleuca) blood. Aging.

[63] Noh JH, Kim KM, McClusky WG, Abdelmohsen K, Gorospe M (2018). Cytoplasmic functions of long noncoding RNAs. WIREs RNA.

[64] Yu W, Huang M, Chen J, Wu S, Zheng K, Zeng S, Zhang K, Hu D (2017). Risk assessment and monitoring of dinotefuran and its metabolites for Chinese consumption of apples. Environ Monit Assess.

[65] Chen Z, Dong F, Li S, Zheng Z, Xu Y, Xu J, Liu X, Zheng Y (2015). Response surface methodology for the enantioseparation of dinotefuran and its chiral metabolite in bee products and environmental samples by supercritical fluid chromatography/tandem mass spectrometry. J Chromatogr.

[66] Chen M, Collins EM, Tao L, Lu C (2013). Simultaneous determination of residues in pollen and high-fructose corn syrup from eight neonicotinoid insecticides by liquid chromatography-tandem mass spectrometry. Anal Bioanal Chem.

[67] Kim D, Langmead B, Salzberg SL (2015). HISAT: a fast spliced aligner with low memory requirements. Nat Methods.

[68] Pertea M, Pertea GM, Antonescu CM, Chang TC, Mendell JT, Salzberg SL (2015). StringTie enables improved reconstruction of a transcriptome from RNA-seq reads. Nat Biotechnol.

[69] Ghosh S, Chan CKK (2016). Analysis of RNA-Seq data using TopHat and cufflinks.

[70] Young MD, Wakefield MJ, Smyth GK, Oshlack A (2010). Gene ontology analysis for RNA-seq: accounting for selection bias. Genome Biol.

[71] Mao X, Tao C, John GO, Wei L (2005). Automated genome annotation and pathway identification using the KEGG Orthology (KO) as a controlled vocabulary. Bioinformatics.

[72] Maciejewski H (2014). Gene set analysis methods: statistical models and methodological differences. Brief Bioinform.

