# Supplementary material for: Identification of long noncoding RNAs reveals the effects of dinotefuran on the brain in Apis mellifera (Hymenopptera: Apidae)
Source: BMC Genomics. 2021 Jul 3;22:502. doi: 10.1186/s12864-021-07811-y (PMC8254963; doi:10.1186/s12864-021-07811-y)
Supplement: Supplementary file 1 — Additional file 1. [file 12864_2021_7811_MOESM1_ESM.pdf]

Additional file 1

Table A1. Summary of the sequencing reads alignment to the reference genome.

Sample	Raw_reads	Clean_reads	Total mapped %	Uniquely mapped %	exonic %	intronic %	intergenic %
DT_1d-1	107967992	106681222	93.80	78.16	81.84	2.37	15.78
DT_1d-2	95760456	94881472	82.35	68.73	80.81	2.64	16.54
DT_1d-3	105161302	104004152	91.28	78.74	82.15	2.47	15.38
C_1d-1	99765664	98205024	90.94	77.95	71.74	4.53	23.73
C_1d-2	105057000	103760336	92.95	76.61	81.15	2.99	15.86
C_1d-3	107693088	106363202	92.30	79.04	82.92	2.76	14.31
DT_5d-1	106591856	104883100	97.18	90.98	84.16	3.32	12.52
DT_5d-2	104737056	103675432	91.02	71.82	79.41	3.25	17.34
DT_5d-3	106869016	105314422	88.43	70.48	81.82	3.44	14.74
C_5d-1	107594942	106295126	89.46	70.52	80.93	3.49	15.59
C_5d-2	107020686	105552092	88.66	67.29	82.55	3.36	14.10
C_5d-3	107293392	106200160	90.26	79.21	75.60	6.54	17.87
DT_10d-1	104524568	103142264	91.46	76.62	78.24	5.34	16.41
DT_10d-2	104165766	102888672	89.90	72.44	76.94	5.34	17.73
DT_10d-3	106231976	104889892	83.80	68.03	76.40	5.94	17.66
C_10d-1	106671238	105113584	93.76	72.75	76.75	3.98	19.27
C_10d-2	104960982	103509828	89.67	67.92	79.72	3.23	17.05
C_10d-3	104122160	102903206	85.15	69.80	80.81	5.32	13.86
