# Supplementary material for: Identification of long noncoding RNAs reveals the effects of dinotefuran on the brain in Apis mellifera (Hymenopptera: Apidae)
Source: BMC Genomics. 2021 Jul 3;22:502. doi: 10.1186/s12864-021-07811-y (PMC8254963; doi:10.1186/s12864-021-07811-y)
Supplement: Supplementary file 2 — Additional file 2. [file 12864_2021_7811_MOESM2_ESM.pdf]

Additional file 2

Table A2. The most abundant known mRNA transcripts in the dinotefuran-treated and control honeybees at different ages

	transcript	gene name	transcript_locus	FPKM
DT_1d	NM_001011582.1	LOC406093	NC_037643.1:15024551-15025504	78699.29
	NM_001011579.1	Mrjp1	NC_037648.1:2274667-2277905	29009.1
	807692.1	ND1	NC_001566.1:12302-15517	12093.95
	NM_001011601.1	Mrjp3	NC_037648.1:2268450-2272030	6906.106
	NM_001011616.2	Defl	NC_037643.1:8673495-8675023	6906.068
	NM_001011580.1	Mrjp2	NC_037648.1:2301571-2304843	6481.375
	XM_006563358.3	LOC113218518	NC_037652.1:5677334-5677995	6463.943
	NM_001011574.1	LOC406081	NC_037642.1:11679274-11685664	6071.255
	NM_001085346.1	Apd-2	NC_037641.1:4488750-4489933	4428.061
	NM_001011583.2	CSP3	NC_037642.1:2779893-2781325	3264.128
C_1d	NM_001011582.1	LOC406093	NC_037643.1:15024551-15025504	55340.14
	NM_001011579.1	Mrjp1	NC_037648.1:2274667-2277905	19434.28
	807692.1	ND1	NC_001566.1:12302-15517	15096.54
	NM_001011615.1	LOC406142	NC_037643.1:2367078-2368717	7013
	NM_001011574.1	LOC406081	NC_037642.1:11679274-11685664	6113.515
	NM_001011616.2	Defl	NC_037643.1:8673495-8675023	5992.357
	NM_001011601.1	Mrjp3	NC_037648.1:2268450-2272030	5559.812
	XM_006563358.3	LOC113218518	NC_037652.1:5677334-5677995	5088.751
	NM_001085346.1	Apd-2	NC_037641.1:4488750-4489933	4957.64
	NM_001011580.1	Mrjp2	NC_037648.1:2301571-2304843	4155.941
DT_5d	NM_001011582.1	LOC406093	NC_037643.1:15024551-15025504	75621.54
	NM_001011601.1	Mrjp3	NC_037648.1:2268450-2272030	8853.043
	NM_001011574.1	LOC406081	NC_037642.1:11679274-11685664	8842.691
	807692.1	ND1	NC_001566.1:12302-15517	6435.248
	NM_001011579.1	Mrjp1	NC_037648.1:2274667-2277905	4493.582
	NM_001011580.1	Mrjp2	NC_037648.1:2301571-2304843	3529.76
	NM_001011616.2	Defl	NC_037643.1:8673495-8675023	3239.057
	NM_001085344.1	Apd-3	NC_037641.1:4479531-4480371	3185.197
	NM_001085346.1	Apd-2	NC_037641.1:4488750-4489933	3168.302
	NM_001011608.1	Hbg3	NC_037643.1:4602439-4613520	2677.496
C_5d	NM_001011582.1	LOC406093	NC_037643.1:15024551-15025504	55166.77
	NM_001011579.1	Mrjp1	NC_037648.1:2274667-2277905	24479.75
	807692.1	ND1	NC_001566.1:12302-15517	13379.97
	NM_001011574.1	LOC406081	NC_037642.1:11679274-11685664	10346.78
	NM_001011608.1	Hbg3	NC_037643.1:4602439-4613520	10346.06
	NM_001085346.1	Apd-2	NC_037641.1:4488750-4489933	6254.554
	NM_001011616.2	Defl	NC_037643.1:8673495-8675023	5430.98
	NM_001085344.1	Apd-3	NC_037641.1:4479531-4480371	3559.691
	807695.1	COX1	NC_001566.1:1794-3424	3293.082
	NM_001011615.1	LOC406142	NC_037643.1:2367078-2368717	2957.637
DT_10d	807692.1	ND1	NC_001566.1:12302-15517	14578.73
	NM_001011582.1	LOC406093	NC_037643.1:15024551-15025504	11859.59
	NM_001011615.1	LOC406142	NC_037643.1:2367078-2368717	8135.89
	NM_001011617.1	LOC406144	NC_037647.1:12267100-12267965	7196.011
	NM_001011583.2	CSP3	NC_037642.1:2779893-2781325	4533.268
	NM_001011616.2	Defl	NC_037643.1:8673495-8675023	3038.455
	NM_001085344.1	Apd-3	NC_037641.1:4479531-4480371	2772.781
	NM_001077820.1	CSP1	NC_037645.1:6453930-6454392	2665.295
	NM_001011574.1	LOC406081	NC_037642.1:11679274-11685664	2645.507
	NM_001085346.1	Apd-2	NC_037641.1:4488750-4489933	2210.542
C_10d	NM_001011582.1	LOC406093	NC_037643.1:15024551-15025504	25201.6
	NM_001011615.1	LOC406142	NC_037643.1:2367078-2368717	18622.69
	807692.1	ND1	NC_001566.1:12302-15517	13127.05
	NM_001011617.1	LOC406144	NC_037647.1:12267100-12267965	9016.143
	NM_001011608.1	Hbg3	NC_037643.1:4602439-4613520	7278.272
	NM_001085346.1	Apd-2	NC_037641.1:4488750-4489933	5214.797
	NM_001011616.2	Defl	NC_037643.1:8673495-8675023	4248.149
	NM_001011574.1	LOC406081	NC_037642.1:11679274-11685664	3609.106
	NM_001011583.2	CSP3	NC_037642.1:2779893-2781325	2592.628
	807695.1	COX1	NC_001566.1:1794-3424	2204.76
