# Supplementary figures and images for: Identification of long noncoding RNAs reveals the effects of dinotefuran on the brain in Apis mellifera (Hymenopptera: Apidae)
Source: BMC Genomics. 2021 Jul 3;22:502. doi: 10.1186/s12864-021-07811-y (PMC8254963; doi:10.1186/s12864-021-07811-y)

Additional file 3

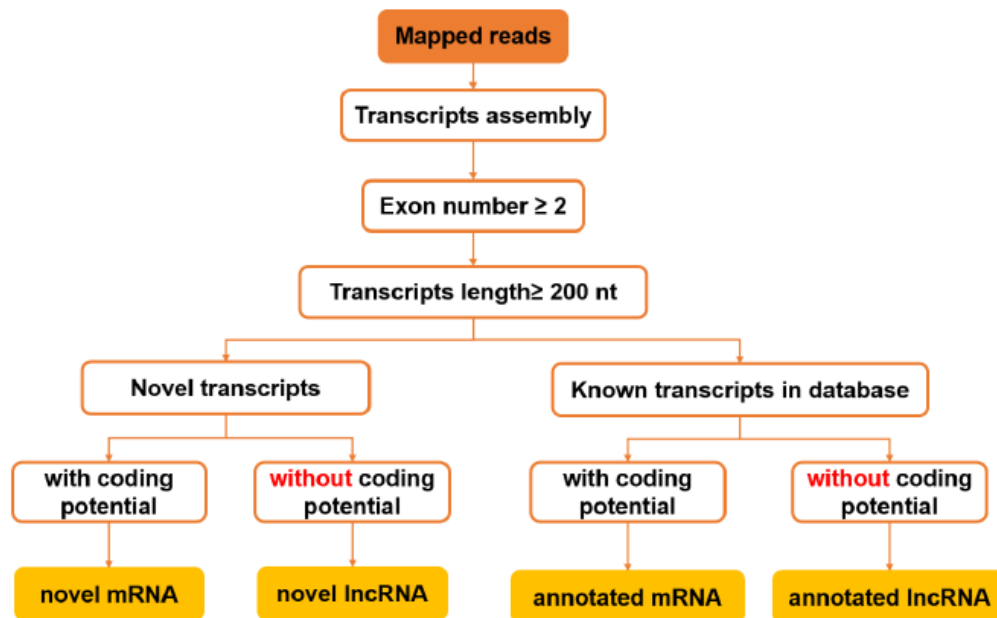

Figure A1: The main workflow for screening lncRNAs.

Supplement: Supplementary file 3 — Additional file 3. [file 12864_2021_7811_MOESM3_ESM.pdf]
