# Supplementary material for: Identification of long noncoding RNAs reveals the effects of dinotefuran on the brain in Apis mellifera (Hymenopptera: Apidae)
Source: BMC Genomics. 2021 Jul 3;22:502. doi: 10.1186/s12864-021-07811-y (PMC8254963; doi:10.1186/s12864-021-07811-y)
Supplement: Supplementary file 4 — Additional file 4. [file 12864_2021_7811_MOESM4_ESM.pdf]

Additional file 4

Table A3. The most abundant lncRNA transcripts in the dinotefuran-treated and control honeybees at different ages.

	transcript	transcript_locus	FPKM
DT_1d	TCONS_00024915	NC_037641.1:11478071-11539732	85532.51302
	TCONS_00086477	NW_020555842.1:14318-25480	11914.64323
	XR_001702285.2	NC_037639.1:11355697-11357162	765.719645
	TCONS_00086824	NW_020555880.1:29114-30149	506.5294393
	TCONS_00086823	NW_020555880.1:29114-30149	455.272868
	TCONS_00030747	NC_037642.1:13064552-13068752	327.236735
	TCONS_00023619	NC_037641.1:4486092-4490026	227.4845647
	TCONS_00028179	NC_037641.1:13154850-13155889	224.2483773
	XR_409884.3	NC_037643.1:7435812-7436865	169.823461
	XR_411275.3	NC_037639.1:15185690-15186784	129.2095287
C_1d	TCONS_00024915	NC_037641.1:11478071-11539732	56428.35547
	TCONS_00086477	NW_020555842.1:14318-25480	12549.70736
	XR_001702285.2	NC_037639.1:11355697-11357162	701.4471537
	TCONS_00086823	NW_020555880.1:29114-30149	606.3378703
	TCONS_00030747	NC_037642.1:13064552-13068752	603.1220247
	TCONS_00086824	NW_020555880.1:29114-30149	284.613912
	TCONS_00028179	NC_037641.1:13154850-13155889	276.4302213
	XR_411275.3	NC_037639.1:15185690-15186784	206.659622
	XR_409884.3	NC_037643.1:7435812-7436865	154.5639217
	TCONS_00023620	NC_037641.1:4486092-4490026	132.3845013
DT_5d	TCONS_00024915	NC_037641.1:11478071-11539732	73165.89583
	TCONS_00086477	NW_020555842.1:14318-25480	11362.77757
	TCONS_00086823	NW_020555880.1:29114-30149	1532.792774
	TCONS_00086519	NW_020555855.1:1-1982	1149.655304
	XR_001702285.2	NC_037639.1:11355697-11357162	669.3395997
	TCONS_00033696	NC_037642.1:13063837-13065881	457.3844403
	TCONS_00086824	NW_020555880.1:29114-30149	303.9292603
	TCONS_00030747	NC_037642.1:13064552-13068752	247.898401
	TCONS_00028179	NC_037641.1:13154850-13155889	236.4205883
	TCONS_00023619	NC_037641.1:4486092-4490026	150.445522
C_5d	TCONS_00024915	NC_037641.1:11478071-11539732	42076.38932
	TCONS_00086477	NW_020555842.1:14318-25480	16426.67611
	TCONS_00086823	NW_020555880.1:29114-30149	2614.529826
	TCONS_00086824	NW_020555880.1:29114-30149	811.730713
	XR_001702285.2	NC_037639.1:11355697-11357162	488.870565
	TCONS_00030747	NC_037642.1:13064552-13068752	466.84671
	TCONS_00028179	NC_037641.1:13154850-13155889	326.6606953
	XR_411131.3	NC_037639.1:11829037-11837782	261.950246
	XR_411275.3	NC_037639.1:15185690-15186784	173.987442
	XR_409884.3	NC_037643.1:7435812-7436865	157.50266
DT_10d	TCONS_00024915	NC_037641.1:11478071-11539732	50053.4974
	TCONS_00086477	NW_020555842.1:14318-25480	15259.15983
	XR_001702285.2	NC_037639.1:11355697-11357162	853.9612223
	TCONS_00086823	NW_020555880.1:29114-30149	843.3205567
	TCONS_00086824	NW_020555880.1:29114-30149	837.546346
	TCONS_00030747	NC_037642.1:13064552-13068752	818.8304037
	TCONS_00028179	NC_037641.1:13154850-13155889	527.3793843
	TCONS_00033696	NC_037642.1:13063837-13065881	426.677624
	XR_411131.3	NC_037639.1:11829037-11837782	277.181371
	XR_411275.3	NC_037639.1:15185690-15186784	258.7188363
C_10d	TCONS_00024915	NC_037641.1:11478071-11539732	62554.24349
	TCONS_00086477	NW_020555842.1:14318-25480	18162.96354
	TCONS_00086823	NW_020555880.1:29114-30149	2190.83374
	TCONS_00030747	NC_037642.1:13064552-13068752	730.8039753
	XR_001702285.2	NC_037639.1:11355697-11357162	725.2678627
	TCONS_00086824	NW_020555880.1:29114-30149	496.0540363
	TCONS_00028179	NC_037641.1:13154850-13155889	441.522237
	XR_411275.3	NC_037639.1:15185690-15186784	276.8747663
	XR_411131.3	NC_037639.1:11829037-11837782	240.3767397
	XR_409884.3	NC_037643.1:7435812-7436865	183.5949403
