# Supplementary material for: Identification of long noncoding RNAs reveals the effects of dinotefuran on the brain in Apis mellifera (Hymenopptera: Apidae)
Source: BMC Genomics. 2021 Jul 3;22:502. doi: 10.1186/s12864-021-07811-y (PMC8254963; doi:10.1186/s12864-021-07811-y)
Supplement: Supplementary file 7 — Additional file 7. [file 12864_2021_7811_MOESM7_ESM.pdf]

Additional file 7

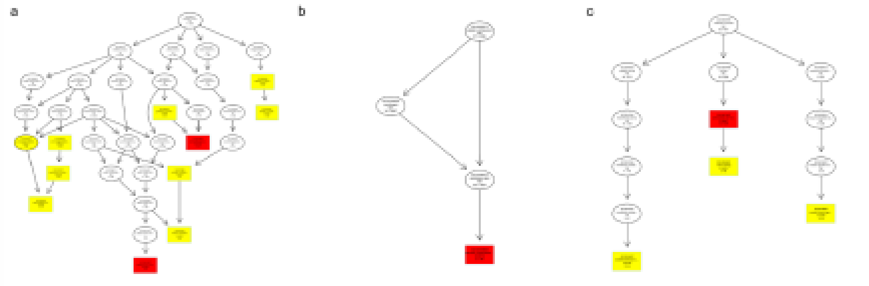

Figure A6. GO categorization of target genes in *trans* regulation of the DE lncRNAs identified in DT_1d vs. C_1d. (a) Biological processes; (b) cellular components; and (c) molecular Functions. The most significant enrichment is indicated by red, followed by yellow. Rectangles represent the top 10 GO terms of enrichment, and circles represent other GO terms.

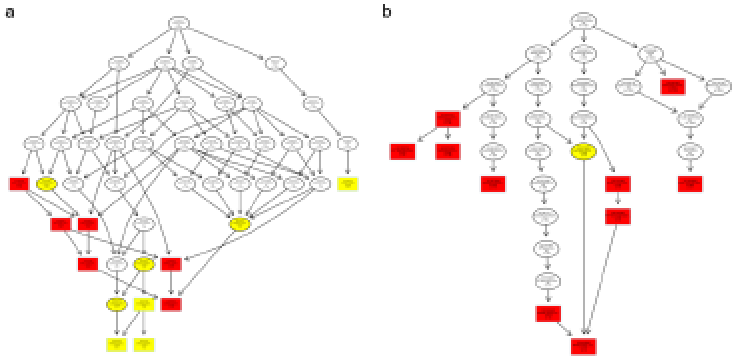

Figure A7. GO categorization of target genes in trans regulation of the DE lncRNAs identified in DT_5d vs. C_5d. (a) Biological processes; and (b) molecular Functions. The most significant enrichment is indicated by red, followed by yellow. Rectangles represent the top 10 GO terms of enrichment, and circles represent other GO terms.

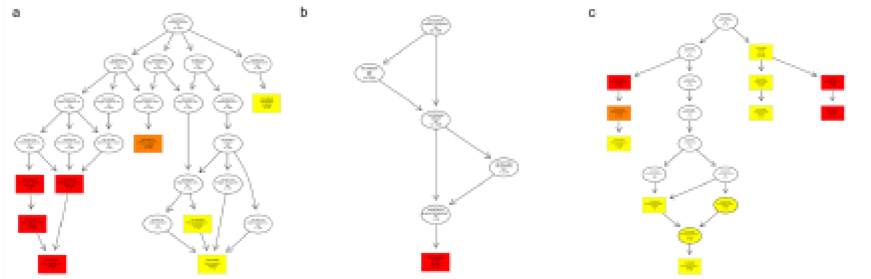

Figure A8. GO categorization of target genes in trans regulation of the DE lncRNAs identified in DT_10d vs. C_10d. (a) Biological processes; (b) cellular components; and (c) molecular Functions.
