# Supplementary material for: Identification of long noncoding RNAs reveals the effects of dinotefuran on the brain in Apis mellifera (Hymenopptera: Apidae)
Source: BMC Genomics. 2021 Jul 3;22:502. doi: 10.1186/s12864-021-07811-y (PMC8254963; doi:10.1186/s12864-021-07811-y)
Supplement: Supplementary file 8 — Additional file 8. [file 12864_2021_7811_MOESM8_ESM.pdf]

Additional file 8

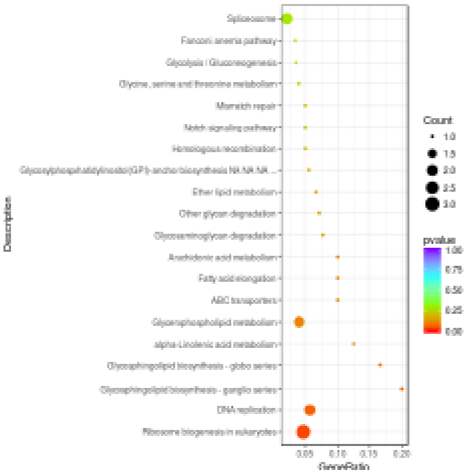

Figure A9. KEGG pathway enrichm9ent analysis of the target genes in *trans* regulation of DE lncRNAs identified in DT_1d vs. C_1d.

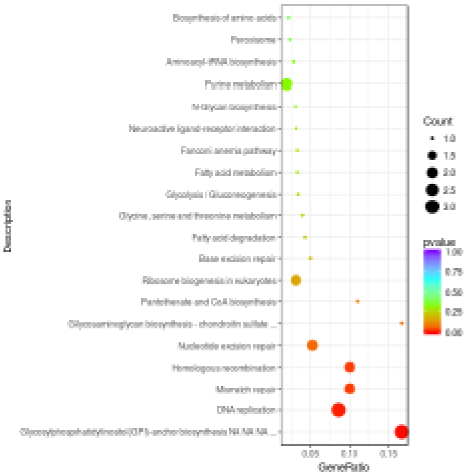

Figure A10. KEGG pathway enrichment analysis of the target genes in trans regulation of DE lncRNAs identified in DT_5d vs. C_5d.

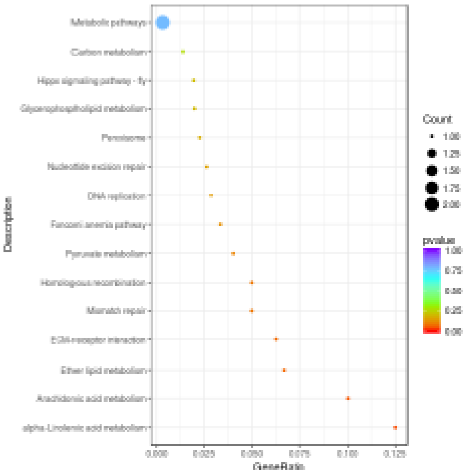

Figure A11. KEGG pathway enrichment analysis of the target genes in trans regulation of DE lncRNAs identified in DT_10d vs. C_10d.
