# Supplementary material for: Identification of long noncoding RNAs reveals the effects of dinotefuran on the brain in Apis mellifera (Hymenopptera: Apidae)
Source: BMC Genomics. 2021 Jul 3;22:502. doi: 10.1186/s12864-021-07811-y (PMC8254963; doi:10.1186/s12864-021-07811-y)
Supplement: Supplementary file 9 — Additional file 9. [file 12864_2021_7811_MOESM9_ESM.pdf]

Additional file 9

Table A4. GSEA enrichment results of dinotefuran-treated and control groups.

Upregulated in class	GS<br>follow link to MSigDB	Pathway	SIZE	ES	NES	NOM p-val	FDR q-val	FWER p-val	RANK AT MAX	LEADING EDGE
DT_1d	AMI00072	Synthesis and degradation of ketone bodies	16	0.5711147	1.749095	0.011407907	0.11127928	0.019	4408	tags=88%, list=44%, signal=157%
DT_1d	AMI00040	Pentose and glucuronate interconversions	24	0.240199	1.35426	0.027027028	0.22027288	0.075	6777	tags=96%, list=68%, signal=296%
C_1d	AMI04391	Hippo signaling pathway	46	-0.49414	-2.158067	0	0.000773718	0.002	3941	tags=63%, list=40%, signal=157%
C_1d	AMI04350	TGF-beta signaling pathway - fly	33	-0.478452	-1.953333	0	0.011457007	0.034	4001	tags=82%, list=40%, signal=136%
C_1d	AMI04341	Hedgehog signaling pathway - fly	24	-0.490312	-1.840981	0.001140531	0.026561452	0.143	4540	tags=75%, list=46%, signal=168%
C_1d	AMI03015	mRNA surveillance	41	-0.393409	-1.802933	0.003003003	0.031710644	0.199	4282	tags=70%, list=43%, signal=122%
C_1d	AMI04013	MAPK signaling pathway - fly	77	-0.384807	-1.780933	0.001	0.031809853	0.234	3720	tags=86%, list=38%, signal=103%
C_1d	AMI04330	Notch signaling pathway	24	-0.474150	-1.778671	0.003224661	0.032750789	0.27	2643	tags=63%, list=27%, signal=85%
C_1d	AMI04512	ECM-receptor interaction	23	-0.468216	-1.71159	0.003189722	0.05047243	0.429	2082	tags=48%, list=21%, signal=61%
C_1d	AMI04320	Dorso-ventral axis formation	23	-0.446290	-1.690312	0.01266489	0.05649002	0.522	3626	tags=74%, list=36%, signal=116%
C_1d	AMI00250	Alanine, aspartate and glutamate metabolism	27	-0.41327	-1.680032	0.021114504	0.08015456	0.659	1145	tags=37%, list=12%, signal=42%
C_1d	AMI04392	Hippo signaling pathway - multiple species	11	-0.477186	-1.583017	0.034820436	0.11761366	0.822	4396	tags=82%, list=44%, signal=155%
C_1d	AMI04144	Endocytosis	113	-0.318752	-1.574804	0.002	0.11710856	0.831	4685	tags=60%, list=47%, signal=120%
C_1d	AMI04310	Wnt signaling pathway	69	-0.354129	-1.548040	0.007007007	0.13376804	0.887	4952	tags=72%, list=50%, signal=145%
C_1d	AMI02010	ABC transporters	24	-0.396872	-1.501468	0.042313374	0.17809011	0.962	3377	tags=67%, list=34%, signal=104%
C_1d	AMI00310	Lysine degradation	39	-0.347319	-1.492023	0.04028978	0.17778766	0.97	4376	tags=62%, list=44%, signal=110%
C_1d	AMI04070	Phosphatidylinositol signaling system	37	-0.350494	-1.476878	0.024096385	0.18480062	0.983	4349	tags=67%, list=44%, signal=122%
C_1d	AMI04120	Ubiquitin mediated proteolysis	83	-0.308733	-1.473634	0.012	0.18081799	0.984	3822	tags=58%, list=39%, signal=93%
C_1d	AMI04068	FoxO signaling pathway	71	-0.323498	-1.425036	0.04112337	0.23040234	0.994	3195	tags=76%, list=32%, signal=109%
C_1d	AMI04150	mTOR signaling pathway	81	-0.299147	-1.406923	0.054	0.2488316	0.998	3395	tags=73%, list=34%, signal=108%
C_3d	AMI00260	Glycine, serine and threonine metabolism	34	-0.577818	-2.264021	0	0	0	2141	tags=59%, list=22%, signal=73%
C_3d	AMI00280	Valine, leucine and isoleucine degradation	33	-0.576676	-2.233166	0	0	0	2050	tags=64%, list=21%, signal=80%
C_3d	AMI00071	Fatty acid degradation	24	-0.612931	-2.199461	0	0	0	2083	tags=75%, list=21%, signal=95%
C_3d	AMI00561	Glycerolipid metabolism	41	-0.513225	-2.158832	0	0.000778086	0.003	2252	tags=66%, list=23%, signal=85%
C_3d	AMI00410	beta-Alanine metabolism	24	-0.56461	-1.997664	0	0.005286447	0.019	1750	tags=58%, list=17%, signal=71%
C_3d	AMI00981	Insect hormone biosynthesis	17	-0.5726	-1.895111	0.005277100	0.008609314	0.065	1758	tags=47%, list=17%, signal=57%
C_3d	AMI04141	Protein processing in endoplasmic reticulum	114	-0.42334	-1.889626	0	0.00805004	0.066	5135	tags=81%, list=52%, signal=167%
C_3d	AMI00640	Propanoate metabolism	28	-0.50147	-1.886059	0	0.007115408	0.066	1750	tags=54%, list=17%, signal=65%
C_3d	AMI00310	Lysine degradation	39	-0.46773	-1.88767	0	0.006771448	0.069	1650	tags=41%, list=16%, signal=49%
C_3d	AMI00650	Butanoate metabolism	17	-0.576604	-1.868882	0.004107209	0.00708479	0.08	1650	tags=60%, list=16%, signal=72%
C_3d	AMI00760	Nicotinate and nicotinamide metabolism	19	-0.553717	-1.853887	0.002096436	0.008107809	0.112	3896	tags=79%, list=39%, signal=130%
C_3d	AMI00040	Pentose and glucuronate interconversions	24	-0.503083	-1.802239	0.002083333	0.00878463	0.114	2349	tags=54%, list=24%, signal=71%
C_3d	AMI00380	Tryptophan metabolism	22	-0.501920	-1.786178	0.006237006	0.01437020	0.194	1741	tags=45%, list=18%, signal=55%
C_3d	AMI00620	Pyruvate metabolism	34	-0.472046	-1.77194	0.00208814	0.01454031	0.22	3323	tags=68%, list=33%, signal=104%
C_3d	AMI00531	Glycosaminoglycan degradation	16	-0.524758	-1.767027	0.01028751	0.01018670	0.281	321	tags=31%, list=3%, signal=32%
C_3d	AMI02010	ABC transporters	24	-0.480498	-1.746133	0.006032447	0.01817779	0.285	2940	tags=67%, list=30%, signal=95%
C_3d	AMI03040	Spliceosome	109	-0.369901	-1.711546	0.001001001	0.02442814	0.365	4800	tags=77%, list=49%, signal=150%
C_3d	AMI00250	Alanine, aspartate and glutamate metabolism	27	-0.45402	-1.696179	0.00814606	0.0304704	0.444	3238	tags=55%, list=33%, signal=103%
C_3d	AMI00230	Purine metabolism	76	-0.361432	-1.664033	0.00208408	0.04121248	0.581	4443	tags=71%, list=45%, signal=128%
C_3d	AMI00053	Ascorbate and aldarate metabolism	18	-0.479015	-1.623523	0.01459724	0.04791331	0.662	2349	tags=50%, list=24%, signal=66%
DT_10d	AMI01040	Biosynthesis of unsaturated fatty acids	32	0.593149	1.856096	0	0.01483934	0.018	1097	tags=41%, list=11%, signal=46%
DT_10d	AMI00010	Oxidative phosphorylation	47	0.533159	1.831961	0	0.00781373	0.019	1180	tags=40%, list=12%, signal=46%
DT_10d	AMI00062	Fatty acid elongation	23	0.592847	1.758963	0.001113586	0.02606828	0.092	1358	tags=44%, list=14%, signal=51%
DT_10d	AMI00190	Oxidative phosphorylation	93	0.424865	1.48964	0.006082119	0.3325488	0.823	4464	tags=78%, list=45%, signal=133%
DT_10d	AMI00051	Fructose and mannose metabolism	30	0.527481	1.487766	0.03424673	0.3018892	0.832	980	tags=30%, list=10%, signal=33%
DT_10d	AMI00020	Citrate cycle (TCA cycle)	36	0.490422	1.470874	0.02310289	0.34926063	0.871	2802	tags=56%, list=28%, signal=77%
DT_10d	AMI00030	Pentose phosphate pathway	22	0.500014	1.45117	0.048478815	0.37911063	0.897	1040	tags=36%, list=11%, signal=41%
DT_10d	AMI00620	Pyruvate metabolism	34	0.456724	1.428814	0.04006329	0.33838857	0.961	2701	tags=50%, list=27%, signal=68%
DT_10d	AMI04070	Phosphatidylinositol signaling system	37	0.411155	1.380493	0.03650116	0.41027102	0.983	3920	tags=41%, list=39%, signal=101%
DT_10d	AMI04080	Neuroactive ligand-receptor interaction	42	0.408137	1.372071	0.02362447	0.39886163	0.987	2203	tags=50%, list=22%, signal=63%
DT_10d	AMI00562	Inositol phosphate metabolism	40	0.41242	1.357774	0.04010345	0.33882374	0.990	3920	tags=50%, list=39%, signal=87%
C_10d	AMI00053	Ascorbate and aldarate metabolism	18	-0.669811	-2.366083	0	0	0	928	tags=56%, list=9%, signal=61%
C_10d	AMI00040	Pentose and glucuronate interconversions	24	-0.497648	-2.085643	0	0.00247477	0.002	928	tags=38%, list=9%, signal=41%
C_10d	AMI00260	Glycine, serine and threonine metabolism	34	-0.426670	-1.892082	0	0.01082677	0.019	858	tags=35%, list=9%, signal=38%
C_10d	AMI00790	Folate biosynthesis	20	-0.582106	-1.46297	0.04950495	0.23807781	0.277	4140	tags=100%, list=42%, signal=[illegible]
