# Supplementary material for: Identification of long noncoding RNAs reveals the effects of dinotefuran on the brain in Apis mellifera (Hymenopptera: Apidae)
Source: BMC Genomics. 2021 Jul 3;22:502. doi: 10.1186/s12864-021-07811-y (PMC8254963; doi:10.1186/s12864-021-07811-y)
Supplement: Supplementary file 10 — Additional file 10. [file 12864_2021_7811_MOESM10_ESM.pdf]

Additional file 10

Table A5. The putative target mRNAs of the lncRNA TCONS_00086519.

mRNA_Gene_ID	mRNA_Gene_Symbol	Pearson_correlation	Pvalue
113219358	LOC113219358	0.951245746	1.38E-09
113218767	LOC113218767	0.993222192	2.19E-16
100578576	LOC100578576	0.955166335	7.13E-10
107964769	LOC107964769	0.993222192	2.19E-16
